# Linkage of Maternal Caregiver Smoking Behaviors on Environmental and Clinical Outcomes of Children with Asthma: A Post-Hoc Analysis of a Financial Incentive Trial Targeting Reduction in Pediatric Tobacco Smoke Exposures

**DOI:** 10.3390/ijerph17228502

**Published:** 2020-11-17

**Authors:** Mandeep S. Jassal, Cassia Lewis-Land, Richard E. Thompson, Arlene Butz

**Affiliations:** 1 Department of Pediatrics, Johns Hopkins School of Medicine, Baltimore, MD 21287, USA; clewis4@jhmi.edu (C.L.-L.); abutz@jhmi.edu (A.B.); 2 Department of Biostatistics, Johns Hopkins Bloomberg School of Public Health, Baltimore, MD 21287, USA; rthomps3@jhu.edu

**Keywords:** asthma, environmental tobacco smoke, secondhand smoke exposure, pediatric, smoking cessation, health promoting financial incentives, cotinine, tobacco

## Abstract

(1) *Background*: Monthly variability in smoking behaviors in caregivers of pediatric asthmatics yields questions of how much and when does smoking reduction result in improved environmental and clinical outcomes. (2) *Methods*: Post hoc analysis of data from a 6 month pilot randomized-control trial occurring from May 2017 to May 2018 in Baltimore City (MD, USA). The initial trial’s primary intervention explored the utility of financial incentives in modifying caregiver smoking behaviors. Post hoc analyses examined all dyads independent of the initial trial’s randomization status. All caregivers received pediatric tobacco smoke harm reduction education, in addition to monthly encouragement to access the state tobacco quitline for individual phone-based counseling and nicotine replacement therapy. Maternal caregivers who were active cigarette smokers and their linked asthmatic child (aged 2–12 years) were grouped into two classifications (“high” versus “low”) based on the child and caregiver’s cotinine levels. A “low” cotinine level was designated by at least a 25% reduction in cotinine levels during 3 months of the trial period; achieving ≤2 months of low cotinine levels defaulted to the “high” category. Twenty-seven dyads (caregivers and children) (total *n* = 54) were assigned to the “high” category, and eighteen dyads (caregivers and children) (total *n* = 36) were allocated to the “low” category. The primary outcome measure was the correlation of caregiver cotinine levels with pediatric cotinine values. Secondary outcomes included asthma control, in addition to caregiver anxiety and depression. (3) *Results*: Caregivers with 3 months of ≥25% decrease in cotinine levels had a significantly greater mean change in child cotinine levels (*p* = 0.018). “Low” caregiver cotinine levels did not significantly improve pediatric asthma control (OR 2.12 (95% CI: 0.62–7.25)). Caregiver anxiety and depression outcomes, measured by Patient Health Questionnaire (PHQ)-4 scores, was not significantly different based on cotinine categorization (*p* = 0.079); (4) *Conclusion*: Reduced pediatric cotinine levels were seen in caregivers who reduced their smoking for at least 3 months, but clinical outcome measures remained unchanged.

## 1. Introduction

Children with asthma in lower-income families are exposed to higher tobacco smoke exposures (TSE) that have been linked with worsened asthma severity [1,2,3]. An estimated one-third of these exposures occur in the home setting of which the primary caregiver is suspected to be a predominant source [4,5]. The primary caregiver who smokes is classically viewed as a primary target of TSE reduction, as evidenced by public health media campaigns detailing the harms of tobacco smoke and by pediatricians who use in-office visits to directly inform caregivers of the link between TSE and asthma [6,7]. Despite these extensive TSE harm reduction efforts and smaller-scale experimental approaches by academic groups under study settings, conventional cigarette smoking among caregivers still remains high among low-income populations [7,8,9,10,11].

The majority of pediatric-centric studies targeting in-home, caregiver-based tobacco smoke reduction have relied on unidimensional or multi-dimensional approaches using behavioral (e.g., motivational interviewing), technological (e.g., air cleaners), TSE education, and/or cessation strategies (e.g., nicotine replacement therapy) [7,12,13,14]. The overwhelming majority of parents enrolled in these studies continued to smoke and children maintained elevated TSE levels [15,16]. Complicating all pediatric TSE reduction studies in lower-income settings is that exposures resulted from multiple sources within the child’s social network (e.g., family members, daycare providers, and community-based locations) [17,18].

Our previous pilot randomized-control study, from which this work was derived, focused on a novel behavioral economic strategy to target select sources of TSE among children with asthma residing in lower-income settings [19]. We decided to use financial incentives based on successful adult-centric studies for promoting smoking cessation. We hypothesized that their potency would be enhanced in lower socioeconomic populations [20,21,22]. Using a community-informed design based on earlier mixed-methodology work, we offered up to $1000 cash for maternal caregivers and a single member of their social network to reduce their cigarette smoking behaviors, contingent on the quantitative reduction of capillary cotinine levels (biomarker of nicotine) [19,23]. We designated a ≥25% reduction in cotinine as meaningful because our belief is that reduction is more likely immediately achievable than abrupt cessation [24,25,26]. Moreover, a 25% reduction could potentially be clinically impactful based on prior smoking intervention studies [25,27]. For example, a 25% reduction in cigarette smoking was associated with decreased chronic pulmonary symptoms (cough and sputum production) [25]. Although not statistically significant, a 25% reduction in daily consumption of cigarettes has been linked with a decreased lung cancer risk [27]. Furthermore, although our intervention was unsuccessful in reducing pediatric cotinine, we appreciated a non-significantly different decline in cotinine levels among maternal caregivers in both control and intervention cohorts. Our work was consistent with past TSE reduction studies that showed control groups also benefited from constant reminders of the importance of smoking reduction, tobacco harm education, and awareness of local cessation resources [15].

This paper describes a post hoc analysis of our earlier work, with an emphasis on environmental and clinical impacts of reducing maternal caregiver cotinine levels. We focused solely on maternal caregiver smoking behaviors because social network members were difficult to retain in the original study due to perceived fragility and variability in social ties [19]. This work also explores the caregiver anxiety and depression levels due to both their association with cigarette usage, and the actual or perceived pediatric asthma control [28,29]. A 3 month reduction in caregiver cotinine levels was selected as meaningful based on clinical outcomes and the belief that if the time of adherence is at least one-half of the total study time, then it could be pragmatic in clinical settings [30,31]. Because adherence to prescribed medications used for chronic diseases is approximately 50%, and one often cited reason for tobacco reduction is mitigation or abatement of chronic disease processes, we used a similar benchmark [30]. We hypothesized that maternal caregivers who achieved a ≥25% reduction in cotinine in at least 3 months of our 6 month trial were linked with children with lower cotinine levels. The objective of this study was to examine the impact of this magnitude and frequency of reduced smoking on pediatric asthma control and caregiver mental health. This work is informative for both academics formulating environmental measures for tobacco-related outcomes and healthcare providers who more frequently interact with caregivers to discuss TSE reduction for betterment of pediatric outcomes.

## 2. Materials and Methods

### 2.1. Study Overview

The methodology, design, and results of the pilot randomized-control trial (RCT) have been previously reported [19]. Briefly, this 6 month RCT compared usual care with an incentive program targeting smoking reduction among adult caregivers of children with asthma and an adult member of their social network. Recruitment of triads began through the identification of children with persistent asthma (as per National Heart, Lung, and Blood Institute criteria) in pediatric pulmonary specialty clinics and inpatient units at Johns Hopkins Children’s Center [32]. We selected the age range (2–12 years old) based on the difficulties of reliably diagnosing asthma below the age of 2 years and the lower likelihood that children above the age of 12 years in our local setting spend significant time indoors with their adult caregivers. We designated salivary cotinine ≥1.0 ng/mL as exposure to tobacco smoke based on previous TSE studies [33,34]. Exclusion criteria among children included the presence of major pulmonary co-morbidities (e.g., bronchopulmonary dysplasia, interstitial lung disease, and recurrent aspiration) that could potentially confound linkage of TSE and pediatric asthma-related outcomes. In addition to the child, their primary maternal caregiver and a member of their social network whose active smoking status was confirmed by capillary cotinine levels were enrolled in the study. We excluded caregivers if they utilized electronic nicotine devices whose usage would erroneously affect the incentive schema, as described below. All study participants received standard TSE education based on Centers for Disease Control and Prevention factsheets and participants received monthly encouragement to use the state tobacco quitline, which had the capacity to offer individual phone-based counseling and up to 4 weeks of nicotine replacement therapy (NRT) [35]. Adult participants in the intervention cohort were eligible for up to $500 over the 6 month study period. Participants received $50 per month for low cotinine levels (>25% decrease compared to baseline values), with an additional $100 given at 3 and 6 months for low cotinine levels during the previous 2 months. Triads enrolled in the control condition received $20 each month as reimbursement for study participation for a total of $120 over the study period. The protocol was approved by the institutional review board at The Johns Hopkins University School of Medicine (IRB00064875). Participants were recruited from May 2017 to May 2018.

#### 2.1.1. Post Hoc Analysis

**Figure 1:**
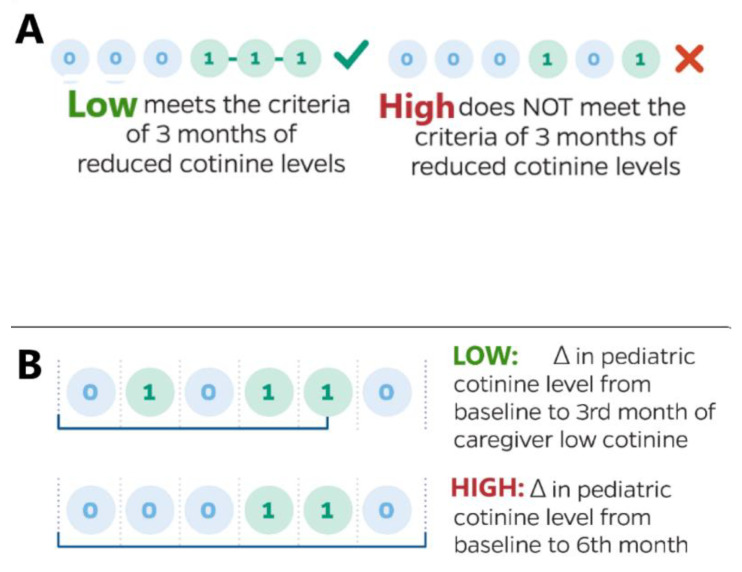
Post hoc analyses overview. Subset (**A**): Schema used to divide caregivers into 2 classifications: “low” versus “high”. Caregivers within the “low” classification had at least three low monthly cotinine levels, compared to baseline (represented by the number “1”). The three low levels do not have to be consecutively observed for classification as “low”; solely 3 low time points need to be noted over the 6 month trial. Achieving ≤2 months of low cotinine levels default to the “high” classification status; Subset (**B**): Measurement schema used to measure the reduction in child cotinine levels. For those caregivers achieving a “low” status, we measured the difference in child cotinine levels at the 3rd time point, compared to baseline values. For those caregivers not designated under a “low” status, we measured the change in cotinine level at 6 months, compared to baseline.

The present post hoc analysis included all asthmatic children and maternal primary caregivers. Social network members were excluded from the current analysis given the inability to retain the majority of the network population for the duration of the RCT. We applied a pattern recognition schema to maternal caregiver monthly cotinine levels, regardless of the initial randomization to the incentive intervention. Caregiver’s were classified through a numerical designation, whereby “1” indicated monthly capillary cotinine levels ≤25% of baseline measures; a value of “0” represented monthly cotinine levels >25% from baseline values. We divided caregivers into 2 classifications: “low” versus “high”. “Low” was defined as a minimum of 3 months of reduced cotinine levels and “high” as all other patterns (see Figure 1).

#### 2.1.2. Endpoints

The primary outcome was the reduction in pediatric cotinine levels at the time when the 3rd “low” month of caregiver cotinine was observed (see Figure 1). Secondary endpoints used caregivers’ classification into “low” and “high” cohorts to assess: (a) correlation of caregiver report of anxiety and/or depression, (b) caregiver nicotine dependence, and (c) child asthma control. The correlations compared outcome measurements at the fulfillment of the 3rd month of low cotinine levels in those classified as “low”, compared to the final 6-month study period in those classified as “high”.

### 2.2. Outcome Measurements

#### 2.2.1. Cotinine

Measurement of capillary cotinine results used PTS© Detect™ cotinine system (PTS Diagnostics©, Whitestown, IN, USA). The cotinine levels were used for biochemical verification of 7 day nicotine reduction [36]. The PTS test provided values ranging from 25–200 ng/mL; values beyond those ranges were designated by the testing device using “<” or “>” symbols. Values < 25 and >200 were defaulted to 24 and 201, respectively, for statistical analyses.

#### 2.2.2. Asthma Control

We measured asthma control in participants aged ≤3 years using the Test for Respiratory and Asthma Control in Kids (TRACK) and aged ≥4 years using the Asthma Control Test (ACT) [37,38]. TRACK is a validated questionnaire to monitor respiratory symptoms control in preschool-aged populations, whereas the ACT has been extensively studied in school-aged children. Asthma control analyses controlled for maternal mental health and nicotine addiction measures. Both measures were used to account for confounding given our initial study’s focus on adult tobacco outcomes and the environmental associations with asthma control.

#### 2.2.3. Caregiver Anxiety and Depression

Anxiety and depression for the caregiver were determined using the Patient Health Questionnaire-4 (PHQ-4) [39]. This brief, 4 item questionnaire has demonstrated reliability and validity in detecting depression and anxiety when a total score of ≥3 has been reported in the two questions relating to each mental health outcome [39]. Individual markers of anxiety and depression were classified as either none/low or moderate/high likelihood; the latter was defined by a total score ≥ 3.

### 2.3. Statistical Analysis

Baseline characteristics of the cohorts were examined using summary statistics (e.g., mean or median). Descriptive statistics used measures of central tendency to describe baseline pediatric asthma control and caregiver mental health (anxiety and depression). Nominal scaling was used to describe caregiver cotinine levels classified as “Low” (0) or “High” (1). We used non-transformed, non-normalized data for generalized estimating equation (GEE) modeling because log-transformation did not alter the significance of the results (Stata Version 15.1, College Station, TX, USA). Wilcoxon rank sum testing compared nominal scaled caregiver cotinine levels and pediatric cotinine levels at two time points: either when caregivers achieved “low” status or at the 6 month time point in those assigned with “high” cotinine status. The correlation between asthma control and achievement of caregiver cotinine levels (high or low status) were investigated by crude odds ratios and adjusted odds ratios. Adjusted odds ratios applied logistic regression modelling using variables previously described as correlates of adult smoking behaviors [40,41]. Confidence intervals (95%) were calculated for both crude and adjusted odds ratios. Pearson correlation coefficient was used for correlation analyses between maternal mental health outcomes and corresponding fulfillment of high or low cotinine status. Due to the relatively low proportion of missing data (10% for children and caregivers), and the possibility that there was not a random loss of the missing data, missing data was not imputed for the analyses. All reported *p*-values are two sided and statistical significance was defined as *p* < 0.05. To detect a mean monthly cotinine difference of 75% (salivary cotinine SD 2.2 ng/mL) difference between groups, with 80% power using a cutoff for statistical significance of 0.05, we required a sample size of at least 45 caregiver–child triads.

## 3. Results

### 3.1. Participant Characteristics at Baseline

All participants identified as either African American or mixed ethnicity that included African-American heritage. Among the “high” cotinine cohort, the median ages of the caregiver and child were 30 and 6 years, respectively; the median ages in the “low” cotinine cohort were 35 years for maternal caregivers and 7 years for children. The “high” cotinine cohort (total *n* = 54) consisted of 27 participants equally distributed among linked dyads of maternal caregivers and children (Table 1). The “low” cotinine cohort (total *n* = 36) consisted of 18 participants within each dyad. The gender distribution of enrolled children was approximately equal and >80% resided in households with an annual income that was below the federal poverty level [42]. Children ≥ 4 years assigned to the “high” cotinine group had a baseline median ACT score indicating adequate asthma control, as opposed to the low-cotinine group that self-identified as having sub-optimal control. Younger children (<4 years) in both cotinine groupings were classified as having mild sub-optimal asthma control based on the TRACK questionnaire.

Greater than 90% of the maternal caregivers were the biological mother. The baseline median PHQ-4 scores among caregivers in both cohorts suggested mild anxiety; however, no indications of depression were present in baseline screening. Caregivers in the “high” cotinine category had a high median level of nicotine dependence, as measured by the Fagerstrom Test for Nicotine Dependence; the “low” cotinine cohort had a borderline moderate to severe level of dependence. Greater than 70% of participants in both groups reported living with at least one smoker. The majority in both groups did not report the enforcement of a home smoking ban, but 25% more patients in the “low” cotinine group did report usage of a ban. High levels of TSE were noted in both cohorts (median levels >5 ng/mL), although baseline median cotinine levels were 2 ng/mL higher in children in the “low” cotinine cohort. Median cotinine levels of the maternal caregivers confirmed the presence of active, daily smoking status (median cotinine >5.0 ng/dL in both “low” and “high” cotinine groups).

### 3.2. Caregiver and Child Cotinine Correlation

**Figure 2:**
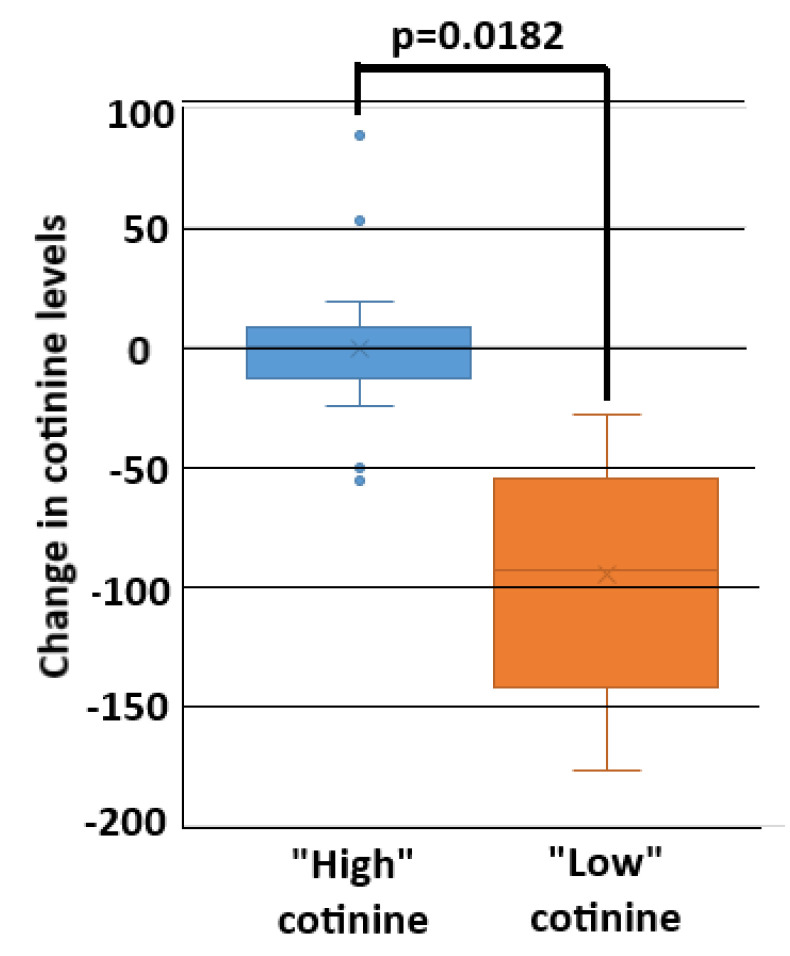
Mean change in pediatric cotinine levels at the time of caregiver fulfillment of “low” cotinine status, compared to the final month in those assigned to the “high” cotinine status.

“Low” cotinine status was attained in 40% of caregivers (Table S1). The lower pediatric cotinine levels were determined at the time when caregivers fulfilled criteria of “low” cotinine status, of which 79% occurred after three consecutive months of low caregiver cotinine levels. The remaining 21% of caregivers achieved a “low” cotinine status after at least one month passing between the first low cotinine value and acquiring two consecutive low cotinine months. Analyses of the change (benchmarked to baseline values) in pediatric cotinine levels at the time that “low” caregiver cotinine status was fulfilled, compared to the change in final pediatric cotinine level for the “high” caregiver cotinine cohort, showed a significant mean decrease in child cotinine levels among those in the “low” cotinine group (*p* = 0.018) (Figure 2).

### 3.3. Asthma Control

Caregivers who had ≥3 months of “low” cotinine levels were not associated with higher reported pediatric asthma control (OR: 2.12; 95% CI: 0.62–7.25, *p* = 0.229) (Table 2). Adjusting for maternal mental health and nicotine addiction measures also did not alter perceived asthma control.

### 3.4. Caregiver Mental Health

**Figure 3:**
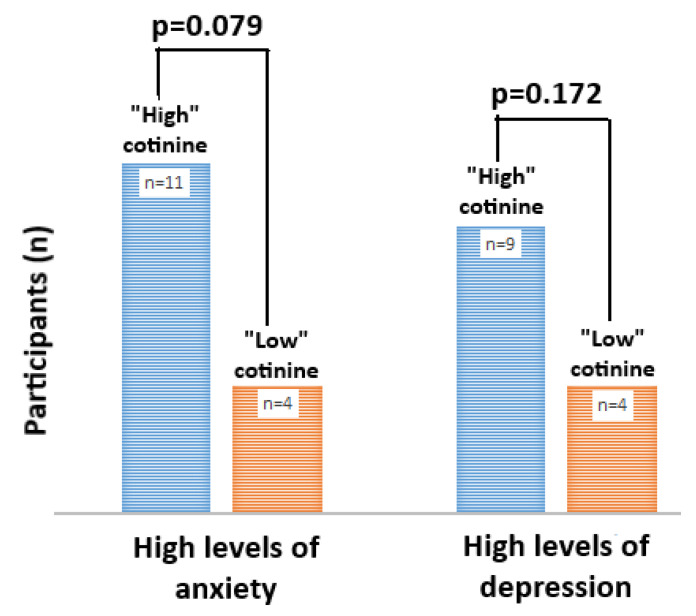
Relationship between caregiver cotinine classification criteria and mental health. Correlation (Pearson χ^2^) of moderate-severe anxiety and depression among caregivers classified by “low”, versus “high” cotinine status.

Although more caregivers within the “high” cotinine cohort had identified themselves with higher levels of anxiety in PHQ-4 scoring, it was not significantly different among those in the “low” cotinine cohort (*p* = 0.079) (Figure 3). The frequency of higher depression was also more notable in the “high” cotinine group, but it was also not significantly different from those assigned to the “low” cotinine cohort (*p* = 0.172).

## 4. Discussion

Our data indicate that a significant reduction in pediatric cotinine levels was achievable among caregivers who could obtain at least 3 months of lower cotinine levels from baseline values. Caregivers classified among the “low” cotinine cohort most frequently achieved their minimum of 3 months reduction of cotinine levels in consecutive months. However, there was no difference in anxiety and depression measures among caregivers in “high” and “low” cohorts. Furthermore, asthma control was not improved in those in the “low” cotinine cohort and correction for caregiver tobacco outcome covariates did not significantly alter the relationship.

This work provides insight into the importance and realities of reducing caregiver smoking behaviors to reduce pediatric cotinine levels. Maternal caregivers overwhelmingly represent the primary individuals who are present for healthcare encounters for their children and thus are an important population for smoking reduction education. Given this reality, maternal caregivers are the recipients of in-person smoking reduction or cessation conversations by pediatric healthcare providers. The remaining smokers responsive for the child’s TSE would ideally undergo secondhand and thirdhand smoke education through mass-education campaigns, and personal communications with the maternal caregiver and/or with their own healthcare provider—all of which have variable efficacy on pediatric environmental outcomes [43,44,45,46]. Our work is specifically informative because select pediatric studies have shown minimal effect on pediatric cotinine levels or nicotine exposure reduction when interventions are primarily targeting adult smoking members of the child’s social network or focused reduction in the primary home setting [47,48]. Future investigations targeting pediatric TSE reduction may benefit from enhanced applications of limited resources to solely the primary maternal caregiver.

Our secondary analyses support the current pediatric dogma that secondhand smoke education and advocacy of parental tobacco smoke reduction should occur at each clinic visit. Caregivers are the most invested entity in their own child’s well-being, and our work supports the time spent by pediatricians for TSE reduction by encouraging cessation contemplation and action [6,49]. Our work shows the value of promoting usage of traditional cessation aids (e.g., nicotine replacement therapies and advocacy of state quitline resources) [6,14,50]. Pediatricians are able to prescribe or assist in acquiring these therapies, which may be beneficial because an active prescriber-based approach can be superior to our study’s passive approach of solely advising participants to self-initiate contact with the state quitline for acquiring nicotine replacement therapy and behavioral counseling.

As evidenced in our trends of reduction of caregiver smoking levels in both cohorts, smoking activities often resume after month(s) of decreased smoking. A return to more intense levels of smoking may be due to high levels of stressors endemic in lower socioeconomic settings in which our patients reside (e.g., financial, housing, food security, and family dynamics) [51,52]. Unfortunately, the limited scope of our project cannot address the multitude of such stressors. Community mental health services were also not optimally accessed by our participants in our original trial based on self-reporting and feedback by our community partners, which may have exacerbated smoking usage among those screening positive for anxiety or depression. Cigarettes in this context may be functioning as anxiolytic or anti-depressive agents [52,53]. Resumption of smoking may also be partly attributed to an altered rate of nicotine metabolism in African-American smokers who may require different forms and frequency of pharmacotherapy regimens [54,55]. We could not sustainably account for these pharmacokinetic discrepancies given the limited duration and types of NRTs that the state quitline could provide. Future studies would benefit from investigator-initiated therapies that are personalized to the individual smoker, including expanding pharmacotherapies to non-nicotine medications (e.g., varenicline or bupropion), in addition to having a longer duration of prescription than what is currently provided by state quitlines (e.g., >1–2 months) [56].

Of note is that this study focused on smoking reduction rather than cessation because reduction is more feasible than abrupt cessation [25,26]. There is insufficient evidence soundly documenting the clinical impact of a 25% reduction in nicotine exposure—especially in terms of pediatric outcomes. Although symptom and disease mitigation has been associated, albeit non-significantly, with a reduction at this level in few adult-based studies, our study would have benefited from utilization of tobacco-linked symptom scoring to document the value of at least a one-quarter reduction in tobacco usage [25,27]. In disease conditions where dose–response relationships among tobacco outcomes are steeper (e.g., chronic obstructive pulmonary disease), it is reasonable to argue that smoking reduction can mitigate some of the harms attributed to high levels of nicotine exposure [57,58]. Moreover, we used a minimum of 3 out of 6 month tobacco reduction as a means to divide cohorts (“high” versus “low”) based on our belief that reduction in tobacco could be equated to prescribed adherence to chronic disease therapy plans (approximately 50%) [30,59]. Because tobacco reduction is often prescribed or recommended by practitioners to reduce the likelihood of chronic disease or symptoms, adherence to smoking reduction should be at least equitable to prescribed medicinal therapies for the same chronic diseases. We also believe our emphasis on reduction could be of particular value to practitioners who engage in personalized conversations with caregivers about modifying tobacco usage. However, our methodology precluded us from firmly concluding if any caregiver achieved cessation due to the limitations in our point-of-care capillary cotinine diagnostic. The lower limit of detection of the testing device was displayed as a cotinine level less than 25 ng/mL, which would not allow for accurately discerning cessation from light active smoking [60,61].

All caregivers were advised to institute a home smoking ban using Centers for Disease Control and Prevention and American Academy of Pediatrics educational materials [35]. This was given greater emphasis on the baseline study visit and was briefly addressed at each follow-up visit. A more intense smoking ban strategy could have been employed, but the initial scope of our study was primarily aimed at the role of predominantly financial incentives in modifying smoking behaviors. Furthermore, we elected not to undertake a more intense approach to promote a home smoking ban based on our earlier mixed methodology work among our target population [23]. The participants enrolled in the study reported smoking bans were not practical for low-income maternal caregivers who did not have the social standing to ask other smokers in the home to modify their smoking behaviors. Moreover, these participants stated that they would prefer resources (e.g., pharmacotherapy and counseling) directed toward the promotion of cessation or reduction, as opposed to smoking bans.

Other limitations of our study include insufficient comparisons of the effect of the intervention compared to pre- and post-intervention time phases. An equal timeline of a comparison of clinical and environmental outcome measures (e.g., 6 months pre- and post-interventions) would provide more meaningful evidence of the long-term and secondary benefits of the intervention. Although missing data was present in 10% of study visits (28 missing data points for children and caregivers among the total of 270 study visits), the missing data could have skewed the results. The missing results would not have impacted the absolute number of those assigned to the “low” cotinine group, but could have altered clinical variables (e.g., asthma control). Missing data could have had some effect on the assignment of those to the “high” cotinine group, whereby the data could have reassigned dyads to the “low” cotinine group—thus, further bolstering our hypothesis. Moreover, we did not incorporate the multitude of clinical and environmental covariates that could potentially influence asthma control. Although we did not see significant odds of improved asthma control with the addition of maternal caregiver-specific covariates (Fagerstrom Test for Nicotine Dependence and mental health), our models would have been more insightful with the incorporation of pediatric-centric asthma outcome measures (e.g., controller medication adherence). Caregiver mental health outcomes used solely the 4 question PHQ to determine anxiety or depression. More comprehensive mental health questionnaires could have yielded a more nuanced understanding of the caregiver mental health, as it relates to the potential usage of cigarettes for its stress reducing or anti-depressive properties. We also did not focus on thirdhand smoke exposures, tobacco-related gasses embedded in materials that can slowly leak into ambient environments, which could have contributed to cumulative pediatric cotinine levels [62,63,64]. Our initial study was focused on secondhand smoke exposures, but the post hoc analyses could have additionally benefited from air nicotine monitoring values or other forms of ambient environmental measures that could elucidate the impact of thirdhand smoke [65]. Lastly, we were unable to assess the influence of feedback of pediatric cotinine levels on caregiver smoking behaviors, mental health, or asthma control outcomes. Pediatric cotinine analyses required multiple weeks for laboratory testing results, which subsequently nullified the accurate assessment of cotinine feedback on immediate caregiver outcomes.

## 5. Conclusions

Pediatric cotinine levels were significantly decreased among caregivers who could reduce their cotinine levels by 25% or greater from baseline levels for at least 3 months. Our study did not conclude that caregiver mental health and pediatric asthma control were affected by the reduction in caregiver smoking patterns, although our analyses were limited by insufficient accounting for the multiple covariates that could influence those outcomes. The demonstration of reduced pediatric cotinine based on the reduction of primary maternal caregiver tobacco reduction may lend further insight for stakeholders looking for a low-cost, high-impact pediatric TSE reduction plan.

## Figures and Tables

**Table 1:** Baseline characteristics of study participants (*n* = 90).

Characteristics	Child (*n* = 45)		Caregiver (*n* = 45)	
	LC (*n* = 18)	HC (*n* = 27)	LC (*n* = 18)	HC (*n* = 27)
Age				
2–4	6 (33%)	8 (30%)		
5–11	11 (61%)	17 (63%)		
12–17	1 (6%)	2 (7%)		
18–30			9 (50%)	6 (22%)
31–50			8 (44%)	20 (74%)
>50			1 (6%)	1 (4%)
Gender				
Male	11 (58%)	12 (44%)		
Female	7 (42%)	15 (56%)	18 (100%)	27 (100%)
Income				
<20 K			14 (79%)	21 (78%)
20–40 k			1 (5%)	6 (22%)
>40 K			2 (11%)	
refused			1 (5%)	
Asthma control *				
TRACK	60	60		
ACT	17	20		
Relationship to child				
Biological mother			17 (94%)	24 (89%)
Biological father				
Maternal grandmother			1 (6%)	2 (7%)
Maternal friend				
Other family				1 (4%)
PHQ-4 *				
Depression			1	2
Anxiety			4	4
FTND *			7	8
Monthly cigarette expenditures ($US)				
<20			2 (11%)	1 (4%)
21–75			12 (67%)	10 (37%)
>76			4 (22%)	16 (59%)
Number of quit attempts in last year				
0			7 (39%)	9 (33%)
1–2			6 (33%)	13 (48%)
>2			5 (28%)	5 (19%)
Methods used to quit in last year				
Abrupt cessation only			7 (64%)	10 (56%)
Behavioral counseling (BC) only				3 (16%)
NRT only			3 (27%)	
Combination of the above			1 (9%)	5 (28%)
Reason(s) for smoking				
Addiction/craving (A)			1 (5%)	
Stress relief (SR)			10 (53%)	11 (41%)
Other			3 (16%)	1 (4%)
A ± SR ± weight control			5 (26%)	15 (55%)
Additional smokers living in home				
0			5 (28%)	6 (22%)
1			13 (72%)	19 (71%)
2				2 (7%)
Indoor home smoking ban				
Yes			8 (44%)	5 (19%)
No			10 (56%)	22 (81%)
Cotinine (ng/mL) *	7.3	5.34	175	192

LC = “low” cotinine levels; HC = “high” cotinine levels; * designated as median value.

**Table 2:** Odds ratio for improved asthma control in participants assigned to the “low”, compared the “high”, cotinine category.

**Asthma control**	**No Adjustment**		**Model 1**		**Model 2**	
	**Crude OR**	**95% CI**	**OR**	**95% CI**	**OR**	**95% CI**
	2.12	0.62–7.25	1.86	0.42–8.15	1.79	0.40–7.89

Model 1: adjusted for anxiety and depression; Model 2: adjusted for model 1 + Fagerstrom.

## Supplementary Materials

The following are available online at https://www.mdpi.com/1660-4601/17/22/8502/s1. Table S1. Monthly cotinine values of maternal caregivers achieving “low” and “high” cotinine classifications. A value of “0” was assigned if the maternal caregiver does not achieve at least three months of “low” cotinine values; a value of “1” was allocated when fulfilling the criteria. Cells without values represent times of inability to acquire monthly cotinine values.
