# Supplementary material for: Linkage of Maternal Caregiver Smoking Behaviors on Environmental and Clinical Outcomes of Children with Asthma: A Post-Hoc Analysis of a Financial Incentive Trial Targeting Reduction in Pediatric Tobacco Smoke Exposures
Source: Int J Environ Res Public Health. 2020 Nov 17;17(22):8502. doi: 10.3390/ijerph17228502 (PMC7696714; doi:10.3390/ijerph17228502)
Supplement: Supplementary file 1 [file ijerph-17-08502-s001.pdf]

<u>Supplementary Table S1</u>. Monthly cotinine values of maternal caregivers achieving "low" and "high" cotinine classifications. A value of "0" was assigned if the maternal caregiver does not achieve at least three months of "low" cotinine values; a value of "1" was allocated when fulfilling the criteria. Cells without values represent times of inability to acquire monthly cotinine values.

"High" cotinine (n=27)							"Low" Cotinine (n=18)						
**Baseline**	**1st month**	**2nd month**	**3rd month**	**4th month**	**5th month**	**6th month**	**Baseline**	**1st month**	**2nd month**	**3rd month**	**4th month**	**5th month**	**6th month**
-	0	0	0	0	0	0	-	1	1	1		1	1
-			0	0	0	0	-	0	1	1	1	1	0
-	0	1	1				-	1	1	1	0	0	1
-				0	0	1	-	0	0	1	1	1	0
-	1	0	0	0		0	-	1	1	1	1	1	1
-	1	0	1				-	0	0	1	1	0	1
-	0	0	0	0	1	0	-	1	1	1	1		1
-	0	0	1	0	0	1	-	1	1	1	1	1	1
-			0		0	0	-	0	1	1	1	1	0
-	0	0	0	0	0	0	-	0	1	1	1	1	1
-	0	0	0	0	0	1	-	0	1	1	1	1	1
-	0	0	0	0	0	0	-	1	0	1	1	0	1
-	1	0	0	0	0	0	-	1	0	1	1	1	1
-	1	0			0	0	-	1	1	0	0	0	1
-	0	0	0	0	0	0	-	1	1	1	1	1	1
-	0		0	0	0	0	-	0	0	1	1	1	1
-		0	0	0	1	0	-	1	1	1	1	1	1
-	0	0	0	0	0	0	-	0	0		1	1	1
-	0	0	0	0	0	0							
-			1	0		1							
-	0	0	0	0	0	0							
-	0	0	0	0	0	0							
-	0	0	0	0	0	0							
-	0	0	0	0	0	0							
-	0	0	0	0	0	0							
-	0	0	0	0	0	0							
-	1				1	0							
